# Chronic Inflammation and the Acceleration of Chronic Disease States

**DOI:** 10.1093/geroni/igab046.1342

**Published:** 2021-12-17

**Authors:** Jeremy Walston

**Affiliations:** Johns Hopkins University School of Medicine, Baltimore, Maryland, United States

## Abstract

The chronic activation of the immune system is commonly observed in older adults, and is highly associated with multiple chronic disease states and Geriatric syndromes including physical frailty, sarcopenia and mild cognitive impairment. Chronic inflammation is multifactorial, and the individual inflammatory mediators that drive the development and propagation of disease states impact normal tissue homeostasis as well as stem cell vitality. This session will discuss age-related etiologies of chronic inflammation and specific inflammatory mediators and their measurement, including Tumor Necrosis Factor (TNF) alpha and its receptors. Inflammation-driven molecular pathways that most impact relevant chronic disease states such as the tryptophan degradation pathway, and its relationship to pathophysiological changes, will also be considered. Finally, discussion of potential treatment modalities, including several emerging from Geroscience research, will be described as will their impact on chronic disease states.

